# Semantic Scene Completion in Autonomous Driving: A Two-Stream Multi-Vehicle Collaboration Approach

**DOI:** 10.3390/s24237702

**Published:** 2024-12-02

**Authors:** Junxuan Li, Yuanfang Zhang, Jiayi Han, Peng Han, Kaiqing Luo

**Affiliations:** 1 Guangdong Provincial Engineering Research Center for Optoelectronic Instrument, School of Electronic Science and Engineering (School of Microelectronics), South China Normal University, Foshan 528225, China; junxuanli@m.scnu.edu.cn (J.L.); hanp@scnu.edu.cn (P.H.); 2 School of Computer Science, Nanjing University of Information Science and Technology, Nanjing 210044, China; 3 Inspur Group, Ji’nan 250000, China; hanjiayi@inspur.com

**Keywords:** semantic scene completion, neighborhood attention transformer, multi-vehicle collaborative perception

## Abstract

Vehicle-to-vehicle communication enables capturing sensor information from diverse perspectives, greatly aiding in semantic scene completion in autonomous driving. However, the misalignment of features between ego vehicle and cooperative vehicles leads to ambiguity problems, affecting accuracy and semantic information. In this paper, we propose a Two-Stream Multi-Vehicle collaboration approach (TSMV), which divides the features of collaborative vehicles into two streams and regresses interactively. To overcome the problems caused by feature misalignment, the Neighborhood Self-Cross Attention Transformer (NSCAT) module is designed to enable the ego vehicle to query the most similar local features from collaborative vehicles through cross-attention, rather than assuming spatial-temporal synchronization. A 3D occupancy map is finally generated from the features of collaborative vehicle aggregation. Experimental results on both V2VSSC and SemanticOPV2V datasets demonstrate TSMV outpace state-of-the-art collaborative semantic scene completion techniques.

## 1. Introduction

Autonomous vehicles (AVs) necessitate precise ambient perception and comprehensive scene understanding to achieve secure and robust autonomous driving [1,2]. Many researchers depict the surrounding environment by using bird’s eye view (BEV) [3,4]. However, the BEV map compresses the height information and limits the fine granularity in the height dimension. Similarly, 3D object detection [5,6] assigns fixed-size bounding boxes to objects, which is difficult to deal with general and extra-lexical objects. Semantic scene completion (SSC) [7,8,9], which can estimate the occupancy state and semantics of the scene, has become an emerging task to overcome these limitations. SSC can provide occupancy for each small unit, rather than assigning bounding boxes, which is conducive to identifying irregular obstacles and less limiting the shape and category of objects. 3D SSC can represent not only the spatial information on the ground plane but also the height dimension, which enhances its capability on rugged roads. Semantic comprehension has a direct impact on subsequent tasks, such as object detection and segmentation [10], which are essential to autonomous driving.

A basic challenge of SSC is to obtain comprehensive information from incomplete observations, often necessitating the model to address occlusion and thoroughly investigate the obscured portion of the scene. Due to the complexity of the occlusion problem, relying solely on single-view sensors may not be the ultimate solution. The existing works [11,12] usually use LiDAR points as input. They aggregate Lidar points from multiple frames to provide rich point cloud information and compensate for long-distance sparsity and partial occlusion problems by adding time-continuous information. However, the fusion of Lidar frames also introduces motion blur, which makes the edges of environmental objects blurred and cannot be accurately identified, suppressing the advantages of a single frame in accurate object positioning. DynStatF [13] proposed a two-stream architecture for the first time to extract features from single-frame and multi-frame LiDAR inputs and interactively fuse them. However, when facing scenes such as intersections, these methods will fail due to the lack of observation from others’ perspectives.

The Internet of Vehicles (IoV) [14] has the potential to ensure driving safety and optimize driving behavior [15]. With the advancement of communication technology, vehicle-to-vehicle (V2V) communication technology [16,17] has gradually matured. Semantic scene completion with V2V provides observation from different perspectives in the same scene which makes it possible to overcome the limitations of a single-vehicle SSC. Recent research for V2V collaboration [18,19,20] exploit the advantages of multiple perspectives. Previous multi-vehicle cooperation works can be divided into three strategies: early, intermediate, and late fusion. Early fusion [21] transmits the original sensor data while late fusion [22] transmits the detection results. The former requires a large bandwidth, and the latter needs additional manual operators to fuse results, such as non-maximum suppression due to the problems of overlap and ambiguity. The fundamental reason for overlap and ambiguity lies in the misalignment of features among cooperative vehicles, leading to boundary ambiguity such as bloated vehicles. This problem is directly reflected in the late fusion and inevitably appears in the other strategies. The last strategy is intermediate fusion, which transmits intermediate features [19,23], performing feature fusion through self-attention. These methods usually have high computational complexity. To solve this problem, self-attention will be restricted to the corresponding position within the feature map [24,25], or the window [26] will be divided to reduce the secondary complexity of attention. However, the features of the corresponding positions of different vehicle feature maps do not strictly belong to the same position.

Therefore, to address the aforementioned issue of the limitation of self-attention and the problem of feature misalignment, we propose a Two-Stream Multi-Vehicle collaboration approach (TSMV), which is the first framework to generate 3D semantic occupancy maps using the collaboration of ego stream and fuse stream. The ego stream pertains to the local environment of the ego vehicle, whereas the fuse stream relates to the comprehensive, interconnected environment of all collaborative vehicles. The two-stream method allows for retaining the relatively accurate environmental boundary information from the ego stream while learning global scene information in the fuse stream, thus alleviating the issue of boundary ambiguity. We believe that the relevant features of the same object should be in the vicinity of the neighborhood. The proposed NSCAT module computes self attention and cross attention by constraining the neighborhood, effectively limiting computational costs while enhancing the features of the two-stream. Our experiments are carried out using the V2VSSC and SemanticOPV2V datasets, and TSMV achieves top performance on collaborative SSC tasks. In conclusion, our work has three primary contributions:

- A novel collaboration approach called TSMV was proposed for collaborative semantic scene completion, which includes a two-stream architecture to effectively fuse features from collaborative vehicles. TSMV alleviates the boundary ambiguity problem caused by feature misalignment in multi-vehicle cooperative perception.
- A NSCAT module was proposed by combining self-attention and cross-attention transformer. NSCAT recurrently aggregates features of two streams through local and global interaction.
- Experiments on both V2VSSC and SemanticOPV2V datasets show that TSMV achieves improved performance compared to the prior arts in both LiDAR-based and Camera-based methods.

## 2. Related Work

### 2.1. Semantic Scene Completion

Semantic occupancy perception originated from SUNCG [27]. The algorithm indicates that learning semantic segmentation and scene completion can be mutually beneficial. Subsequently, more and more attention has been paid to the work of indoor small-scale fixed scenes, such as NYUCAD [28], ScanNet [29] and PALNet [30]. With the success of using dense point clouds or depth maps in small indoor scenes on SSC tasks, researchers have begun to explore dynamic large-scale outdoor scenes. SFSU [31] introduces semantic foggy scene understanding into foggy driving scenes. Ref. [32] proposes an anisotropic network, which decomposes 3D convolution into three consecutive dimensional convolutions, and further enhances modeling capability by stacking multiple kernel-size selection convolutions and kernel modulation convolutions. SemanticKITT [33] extends occupancy perception to driving scenarios, but it only evaluates front-view occupancy prediction. Towards a comprehensive study of surrounding perception algorithms, Openccupancy [34] proposes the first surrounding SSC benchmark. Furthermore, A cascade occupancy network was proposed to refine the coarse prediction dealing with the complexity of high-resolution 3D predictions. SSCBench [35] integrates well-known datasets (e.g., KITTI-360, nuScenes, and Waymo) in autonomous driving to generate SSC benchmarks. V2VSSC [25] is a large-scale V2V 3D semantic occupancy dataset collected by CARLA and collaborative driving automation tool OpenCDA [36]. It can provide observations of multiple vehicle perspectives in the same scene from the simulation server, making up for the limitations of single-view semantic occupancy perception. SemanticOPV2V [37] equips vehicles with semantic LiDAR to obtain 12 different 3D semantic occupancy labels.

### 2.2. Multi-Vehicle Perception

Sharing collective perception messages [38] including GPS and sensor data (lidar, camera, etc.) between vehicles helps to make up for the limitations of current visual perception technologies, such as limited resolution, weather effects, and occlusions. AVs can exchange traffic information with surrounding cooperative vehicles to achieve cooperative perception and improve the safety and robustness of autonomous driving systems. Ref. [39] proposed a novel neural network V2VNet using a spatial perception map for multi-vehicle feature aggregation. FCooper [40] uses a simple max-out operation to fuse features. OPV2V [24] fuses all the features by point-to-point attention at the same location in the feature map. DiscoNet [41] uses knowledge distillation to limit the corresponding features to enhance training. Ref. [42] applies an adaptive feature-level fusion model to fuse multi-vehicle data in simultaneous localization and mapping tasks. V2VFomer++ [43] construct the first multi-model multi-vehicles cooperative perception framework, where individual camera-LiDAR representation is incorporated with dynamic channel fusion module at BEV space. EdgeCooper [44] combines the individual views of the vehicle to form an overall view with higher resolution and proposes a two-dimensional graph coloring algorithm to eliminate conflicts. CoBEVT [45] proposed fused axial attention (FAX) to capture local and global information between different vehicles. V2XViT [26] proposed a novel heterogeneous multi-agent self-attention module (HMSA), which can adaptively integrate heterogeneous agents, such as vehicles and vehicles or vehicles and infrastructure. DAIR-V2X [46] proposed a Time Compensation Late Fusion (TCLF) framework to alleviate the problem of time asynchrony. Lu et al. [47] proposed a scalable heterogeneous cooperative sensing framework Heterogeneous Alliance (HEAL), which uses multi-scale foreground perception to establish a unified feature space.

### 2.3. Transformer in Feature Fusion

Transformer was first proposed for machine translation [48] to capture remote interactions between words by stacking multi-headed self-attention and feed-forward layers. MorphText [49] creates reliable connections between text segments. Dos. A. et al. [50] proposed ViT for image recognition tasks, which directly applies self-attention by cutting image blocks and adding position embedding. The Transformer architecture’s ability to fuse features from different sources or patterns has been verified in previous work. Emerging work series [51,52] integrates multi-modal fusion of images and texts. TransFusion [53] proposed a two-stage Transformer framework. The first stage uses LiDAR features to obtain a set of sparse object queries, and the second stage uses another transformer structure to obtain cross-attention of camera and lidar data. The deformable DETR [54] extracts the cross-attention of the current frame and the previous historical frame. Li et al. [4] used deformable DETR to obtain spatial-temporal attention between multiple images. Although it has global interaction ability, the computational complexity is linearly related to image resolution, and high-resolution images will lead to a quadratic increase in model complexity and memory usage. To solve this problem, many methods introduce locality into self-attention. In SASA [55], each pixel only focuses on the self-attention of a window around it. Swim [56] proposed W-MSA and SW-MSA modules to reduce the secondary complexity and achieve global information interaction. The W-MSA module reduces the amount of calculation by dividing the window, and the SW-MSA solves the problem that information cannot be exchanged between different windows by shifting the window. CSwin [57] proposed a cross-shaped window attention to achieve parallel computing of horizontal and vertical self-attention. Ali et al. [58] proposed a flexible and efficient explicit sliding window attention mechanism, called neighborhood attention. It locates the attention range of each pixel to its nearest field. DynStatF [13] uses a neighborhood attention mechanism to capture the cross-attention of multi-frame and single-frame LiDAR. Different from the previous work, to preserve the spatial correlation, our approach uses the transformers of neighborhood attention to obtain the cross-attention between the LiDAR input features of the cooperative vehicles and the ego vehicle. CoHFF [37] achieves advanced camera-based collaborative semantic occupancy prediction technology through feature sharing in V2X communication networks.

## 3. Methodology

### 3.1. Overall Architecture

**Figure 1:**
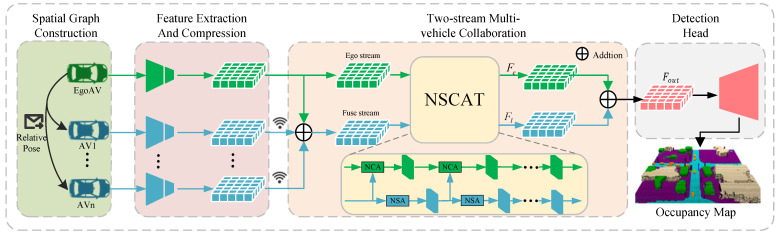
The system framework of TSMV. It consists of four steps: spatial graph construction, feature extraction and compression, two-stream multi-vehicle collaboration, and the detection head. The details of each component are illustrated in Section 3.

The overall system framework of TSMV is illustrated in Figure 1. First, a spatial graph will be constructed on the ego vehicle using V2V communication technology with surrounding vehicles. Following the voxel-based LiDAR detector, we extract the features of the vehicles in the spatial graph and transform them to voxel spatial features $F_{v}$. Then the voxel spatial features are compressed using 3D convolution. The height dimension is compressed to the channel dimension and voxel space features $F_{v}$ is converted into intermediate features $F_{m}$. Subsequently, the nearby AVs broadcast intermediate features $F_{m}$ using the designated method for sharing messages. When the ego vehicle receives the broadcast information, features of all vehicles in the spatial graph will be divided into ego stream and fuse stream. Feature fusion between two streams occurs regressively using the neighborhood self-cross attention module (NSCAT) in our TSMV. Finally, the detection head performs a series of lightweight convolutional operations and applies a reshape operation along the channel dimension to restore the height dimension from the channel dimension. The 3D semantic occupancy map is generated by the detection head.

#### 3.1.1. Spatial Graph Construction

The role of the space graph is to transmit extrinsic and relative positions to each cooperative vehicle. Since the vehicle is moving, the spatial graph will be updated dynamically in real-time. For an ego-vehicle, it searches N∈[1,7] neighboring vehicles within the communication range 70 m as the center around and constructs a spatial graph. Each vehicle within the communication range is represented by a node in the spatial graph, with each communication channel connecting the ego and collaborative nodes being represented by an edge. The node will be deleted from the spatial graph when it exceeds the communication range. On the contrary, when the node enters the communication range, it will be added to the spatial graph to realize the dynamic perception of nearby vehicles by ego vehicle.

#### 3.1.2. Feature Extraction and Compression

The point clouds of neighboring vehicles are projected into the LiDAR coordinate system of the ego vehicle using shared location information. Following the voxel-based LiDAR detection setting, we use VoxelNet [59] to extract features. The projection point clouds will be converted to voxel space features, represented as $F_{v}\in R^{C\times D\times H\times W}$, in which *C* represents the dimension of voxel feature, D/H/W represents the size of voxel grid map along z/y/x direction. The transmission bandwidth of hardware imposes limitations on V2V communication. Due to the high bandwidth requirements for transmitting 3D high-dimensional feature maps, it is essential to compress these feature maps. To further reduce computational burden and the pressure of communication bandwidth, inspired by [60], we use 3D convolution to compress $F_{v}$ and concat the height dimension to the channel dimension, which $F_{m}\in R^{C^{\prime }\times H\times W}$. The compression of height information into channel dimensions can be viewed as a form of BEV which reduces the communication delay and calculation. The compressed intermediate features $F_{m}$ of all vehicles will be fed into the two-stream multi-vehicle collaboration module for fusion, which outputs the final aggregated features $F_{out}$. The two-stream multi-vehicle collaboration module is introduced in Section 3.2.

#### 3.1.3. Head and Losses

The aggregated features $F_{out}$ will be fed to the detection head to generate occupancy probability and semantic category scores. Similar to the Unet structure, we perform a series of lightweight convolutional operations on $F_{out}$, which include several downsampling convolution and upsampling deconvolution layers. Inspired by FlashOcc [60], we perform a simple reshaping operation in the channel dimension to transform the feature map from shape B,C,H,W to $B,\;C^{*},\;Z,\;H,\;W$, where *B* represents batch size, *C* represents channel number in $F_{out},\;Z,H,W$ represent the resolutions in the z/y/x dimensions of 3D space, $C^{*}$ is set to 1 in occupancy prediction, while to the number of semantic category in semantic prediction and $C=C^{*}\times Z$. Finally, we restore the height dimension from the channel dimension and output the final 3D semantic occupancy map.

To train the proposed approach, we leverage geometric loss $L_{g}$ and semantic loss $L_{s}$ to optimize the network. The geometric loss is computed using binary cross-entropy (BCE) loss, indicating whether the voxel position is occupied, while the semantic loss is calculated using cross-entropy (CE) loss, representing whether each unit of voxel is correctly classified semantic category. The general loss function can be derived as:

(1) $$\begin{array}{cc} L & =\lambda_{g}L_{g}+\lambda_{s}L_{s} \end{array}$$

where $\lambda_{g}$ and $\lambda_{s}$ represent the weight of geometric loss and semantic loss respectively.

### 3.2. Two-Stream Multi-Vehicle Collaboration

The fusion of intermediate features from multi-vehicle requires local and global interactions across the spatial location. We divide the intermediate feature of the ego vehicle and the received vehicles’ intermediate features into two streams, called ego stream and fuse stream. The ego stream contains only the intermediate feature of the ego vehicle, denoted as $F_{e}\in R^{C^{\prime }\times H\times W}$. Given the input intermediate features $\left\{F_{m}^{i}|i\in 1\cdots N\right\}$ in the spatial graph, N represents the total number of vehicles, i represents the index of vehicles in the spatial graph, especially $F_{m}^{1}$ represents $F_{e}$. Formally, let $F_{g}\in R^{N\times C^{\prime }\times H\times W}$ be the stacked intermediate features from N vehicles. We map $F_{g}$ to fuse stream features $F_{f}\in R^{C^{\prime }\times H\times W}$ by N-dimensional addition:

(2) $$\begin{array}{cc} F_{f} & =add(F_{g},\;dim=N) \end{array}$$

where add represents features addition. Then, TSMV utilizes the Neighborhood Self-Cross Attention Transformer module (NSCAT) to regress features from the ego local stream to learn features from the fuse global stream. This module is inspired by [13,45,58] and combines self-attention and cross-attention within local windows to enhance features. In contrast to V2VSSC, which applies self-attention to regions at the same position in space to capture representative features. However, simply weighting features of the same spatial position will greatly limit spatial correlation. When faced with issues such as sensor noise, communication delays, localization errors, etc., leading to performance decline. To preserve spatial correlations, we believe that relevant features of the same environmental object should be located in similar positions and neighboring regions in a two-stream features map. Therefore, we establish cross-attention in the intermediate feature for local information nearing a specific query. Furthermore, the intermediate feature map for segmentation tasks is relatively large, limiting the field size helps save computational costs. We apply neighborhood attention(NA), which is a restricted self-attention method used in image classification. Compared to attention methods like Swin, NA achieved efficient GPU acceleration and ran faster while using less memory. Moreover, we extend NA from self-attention to cross-attention to gather information in the local perception field.

**Figure 2:**
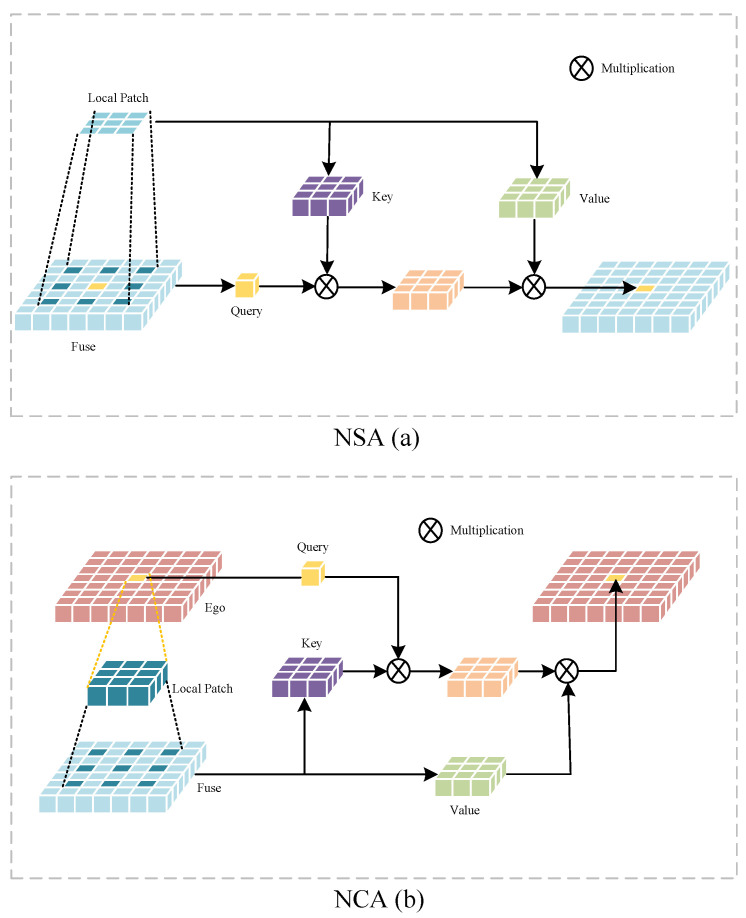
Neighborhood Self Attention (NSA) module (**a**) and Neighborhood Cross Attention (NCA) module (**b**). We use the dilation parameters (control the spacing between the kernel points), where the dilation is 2 in both the NSA and the NCA module.

Neighborhood Self-Attention (NSA) and Neighborhood Cross-Attention (NCA) can be represented as:

(3) $$\begin{array}{c} \left\{\begin{array}{c} Q_{f}=X_{f}W^{q},K_{f}=X_{f}W^{k},V_{f}=X_{f}W^{v}\\ NSA=s\left(\frac{Q_{f}K_{f}^{T}}{\sqrt{v}}\right)V_{f} \end{array}\right. \end{array}$$

(4) $$\begin{array}{c} \left\{\begin{array}{c} Q_{e}=X_{e}W^{q},K_{f}=X_{f}W^{k},V_{f}=X_{f}W^{v}\\ NCA=s\left(\frac{Q_{e}K_{f}^{T}}{\sqrt{v}}\right)V_{f} \end{array}\right. \end{array}$$

where *X* represents neighborhood token features and subscripts represents different streams, *W* represntes trainable parameter matrix, *v* represents feature dimension scale, *s* represents softmax. Specifically, taking the extended neighborhood cross-attention as an example, the convolutional layer is first used to mark the intermediate feature map, which is expressed as a token of m-dimensional feature vectors. For example, the token feature for the ego stream can be expressed as $X_{e}^{token}\in R^{n\times m}$. Token Feature $X_{e}^{token}$ in ego stream are linearly projected to the query $Q_{e}\in R^{n\times q}$, while the token features in the fuse stream are also linearly projected to the key $K_{f}\in R^{n\times q}$ and value $V_{f}\in R^{n\times v}$. For the *i*-th token, the attention weight with neighborhood size of k$\left(A_{i}^{k}\right)$ is:

(5) $$A_{i}^{k}=\left[\begin{array}{c} Q_{e}K_{f}^{\rho 1{\left(i\right)}^{T}}+B_{\left(i,\rho 1(i)\right)}\\ Q_{e}K_{f}^{\rho 2{\left(i\right)}^{T}}+B_{\left(i,\rho 2(i)\right)}\\ \vdots \\ Q_{e}K_{f}^{\rho k{\left(i\right)}^{T}}+B_{\left(i,\rho k(i)\right)} \end{array}\right]$$

where ρj(i) represents the *j*-th nearest neighbor of the *i*-th token, $B_{\left(i,\rho j(i)\right)}$ represents relative positional biases between *j*-th nearest neighbor and *i*-th token. The attention value can be expressed as:

(6) $$V_{i}^{k}=\left[\begin{array}{cccc} V_{f}^{\rho 1{\left(i\right)}^{T}} & V_{f}^{\rho 2{\left(i\right)}^{T}} & \cdots & V_{f}^{\rho k{\left(i\right)}^{T}} \end{array}\right]$$

so the *l*-th token cross attention is defined as:

(7) $$\begin{array}{cc} NCA_{k}\left(i\right) & =\frac{softmax(A_{i}^{k})}{\sqrt{v}}V_{i}^{k} \end{array}$$

where *v* represents the feature dimension scale. We compute the cross attention for each pixel according to the method outlined previously. Similarly, the pixel-by-pixel fuse feature can be obtained by calculating the self-attention of QKV from the fuse stream. The illustration of the self and cross attention are shown in Figure 2.

**Figure 3:**
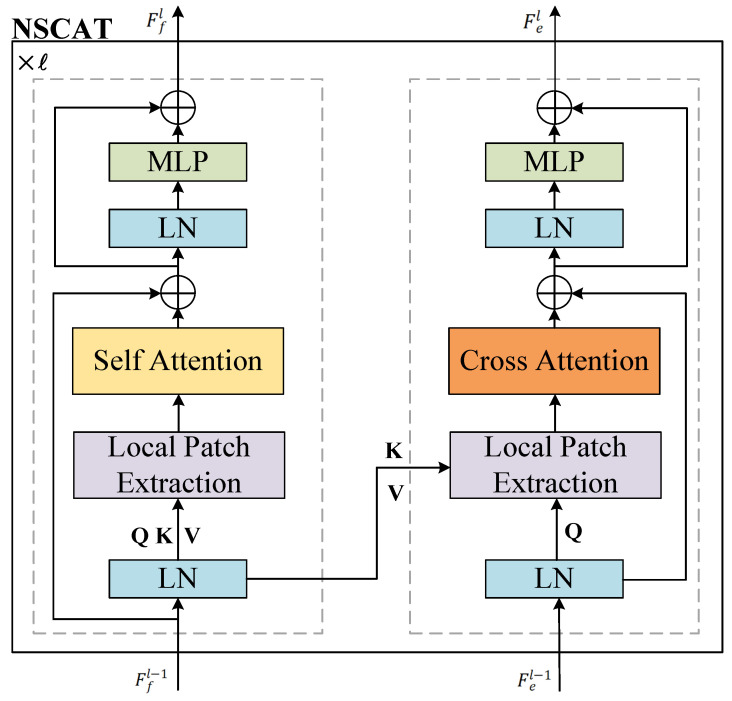
Architectures of Neighborhood Self-Cross Attention Transformer (NSCAT).

We combine two kinds of attention with the classic design of Transformers, including Layer Normalization (LN), Multilayer Perceptrons (MLPs), Feedforward Neural Networks (FFN), and skip-connections as exemplified in Figure 3. In the *l*-th block (l∈1,2,...,L), given the intermediate feature from ego stream $F_{e}\in R^{C\times H\times W}$ and feature from fuse stream $F_{f}\in R^{C\times H\times W}$, the output of the NSCAT module can be expressed as:

(8a) $$\begin{array}{cc} {\hat{F}}_{f}^{l} & =\alpha^{l}\left(LN\left(F_{f}^{l-1}\right)\right)+F_{f}^{l-1} \end{array}$$

(8b) $$\begin{array}{cc} {\hat{F}}_{e}^{l} & =\beta^{l}\left(LN\left(F_{e}^{l-1}\right),LN\left(F_{f}^{l-1}\right)\right)+F_{e}^{l-1} \end{array}$$

(8c) $$\begin{array}{cc} F_{f}^{l} & =MLP\left(LN\left({\hat{F}}_{f}^{l}\right)\right)+{\hat{F}}_{f}^{l} \end{array}$$

(8d) $$\begin{array}{cc} F_{e}^{l} & =MLP\left(LN\left({\hat{F}}_{e}^{l}\right)\right)+{\hat{F}}_{e}^{l} \end{array}$$

where $\alpha^{l}\left(\cdot \right)$ represents NSA module and $\beta^{l}\left(\cdot \right)$ represents NCA module. After all L numbers of NSCAT blocks, we add the intermediate features of the two streams together to sufficiently consolidate features.

Considering that the intermediate features from the ego stream provide accurate environmental perception, while the fuse stream provides rich long-range perception and environmental perception of partially occluded regions from the ego stream, the NSCAT module utilizes the features in ego stream $F_{e}^{l}$ as queries, generating keys and values from fuse stream feature $F_{f}^{l}$ to achieve cross attention and enhance the fuse stream features $F_{f}^{l}$ using the neighborhood self-attention to optimize of the feature map through collaboration. Finally, the outputs of the NSCAT module are added to obtain the final features $F_{out}$, and $F_{out}$ is fed into the detection head.

## 4. Experiments

### 4.1. Datasets and Evaluation Metrics

We conduct experiments on two challenging collaborative autonomous driving datasets, namely V2VSSC dataset and SemanticOPV2V dataset. Both datasets are the extension of the groundbreaking OPV2V dataset [24] which contains over 70 different scenarios. In each scenario, different numbers (2 to 7) of vehicles appear at the same time. Therefore, both datasets are the multi-vehicle collaborative SSC dataset with 6764 training sequences, 1980 validation sequences and 2170 test sequences.

The V2VSSC dataset [25] is a LiDAR-based multi-vehicle collaborative SSC dataset. Each sample consists of a LiDAR sensor and a GNSS/IMU sensor. For the SSC task, the V2VSSC dataset procures precise occupancy and semantic details directly from the server. There are 10,914 annotated semantic occupancy maps with 6 different semantic labels. For the evaluation metrics, The V2VSSC dataset utilizes Intersection of Union (IoU) [33] as the geometric metric, the mean IoU (mIoU) overall categories are utilized as the semantic metric. The IoU and mIoU can be derived as:

(9) $$\begin{array}{cc} IoU & =\frac{TP_{\circ }}{TP_{\circ }+FP_{\circ }+FN_{\circ }} \end{array}$$

(10) $$\begin{array}{cc} mIoU & =\frac{1}{C}\sum_{c=1}^{C}\frac{TP_{c}}{TP_{c}+FP_{c}+FN_{c}} \end{array}$$

where TP,FP,FN correspond to the number of true positive, false positive, and false negative predictions, the subscript ∘ is denoted for occupied voxels while c for categories respectively. In addition, the critical objects of autonomous driving encompass road and vehicles. Therefore, cIoU is defined as the mean of these two categories.

The SemanticOPV2V dataset [37] is a Camera-based multi-vehicle collaborative SSC dataset. Each sample consists of RGB images from 4 cameras with $360^{\circ }$ horizontal FOV and a GNSS/IMU sensor. SemanticOPV2V equips each CAV with 4 semantic LiDARs and associates the ground truth data of all CAVs to capture an accurately collaborative semantic ground truth, thus integrating 12 different 3D semantic occupancy labels. We utilize the metric Intersection Over Union for the evaluation of each category and the mean Intersection Over Union (mean IoU) on the SemanticOPV2V dataset.

### 4.2. Implementation Details

Following previous methods [25,37], we adopt Voxelnet [59] as LiDAR-based backbone and ResNet101 [61] as Camera-based backbone. For experiments on V2VSSC, the perception ranges are [−50 m, 50 m] for the *x*, *y* axis and the size of resolution of the occupancy map is 0.78 m while the perception range of height is [−3 m, 5 m] with the resolution of 0.4 m, so the occupancy map resolution is 20×128×128. We adopt Adam optimizer [62] with an initial learning rate of $2\times 10^{-3}$ and employ cosine anneal warm learning rate scheduler with an initial learning rate of $2\times 10^{-4}$, $\eta_{min}=2\times 10^{-5}$. We conduct training for all models with 30 epochs. Besides, we set up two evaluation models, (1) Perfect setting, in which the positioning is accurate, and all transmission data is synchronized and real-time. (2) Noisy setting, considering the positioning inaccuracies and time delay. Specifically, following the real-world noise levels [63,64], the positional and heading noise are taken from the Gaussian distribution with a default standard deviation of 0.2 m and $0.4^{\circ }$ respectively. In addition, the time delay of all evaluation models is set to 100 ms which reflects the usual transmission delay in V2V communication system [65].

For experiments on SemanticOPV2V, we utilize a 40×40×3.2 m detection area with a grid size of 100×100×8, resulting in a voxel size of 0.4 m^3^. We add the NSCAT module to the V2X feature fusion module in CoHFF [37] to implement a camera-based TSMV method and we set the share features with a length of 64 for Feature Fusion. Following the settings in CoHFF [37], we pretrained Occupancy Network, Semantic Segmentation Network and finally trained Hybrid Feature Fusion Network with pretrained network.

### 4.3. Comparison

#### 4.3.1. LiDAR-Based Results on V2VSSC

For multi-vehicle perception tasks, we compare our proposed model with no-fusion baseline, early and late fusion, as well as various state-of-the-art intermediate fusion LiDAR-based models including FCooper [40], V2VSSC [25], and V2XViT [26].

In Table 1, we report the results on the V2VSSC test set in perfect settings. Under perfect setting, all collaboration methods surpass the no-fusion method, which proves the effectiveness of multi-vehicle collaboration in SSC tasks. Among all fusion models, TSMV outperforms all other methods in IoU, mIoU, and cIoU metrics, while achieving the best in most categories, which demonstrates the effectiveness of TSMV in occupancy and semantic prediction. Specifically, for the comprehensive metric mIoU, it surpasses the second-best by 2%. Moreover, it outperforms the second-best by 2.2% in the car category metric that is most relevant to autonomous driving, with a 2.9% higher accuracy in the cIoU metric including both road and car metrics.

We also conduct experiments under noisy settings on V2VSSC, as shown in Table 2. We fine-tuned the models from the perfect setting. Considering position errors and time delays, the effectiveness of all fusion models shows a decline. The performance of the late fusion method shows a sharp decline, even worse than the no-fusion method. Specifically, for the IoU and mIoU metrics, except for the late fusion method, all other methods are ahead of no-fusion, with TSMV remaining the best performance. Due to AVs being in highly dynamic environments, there was a significant performance decrease across all models in the cIoU metric. Except for TSMV maintaining a lead of 4.4%, the performance of the other methods was either comparable to or worse than the no-fusion baseline method, which proves that our proposed method has certain anti-interference capability and is applicable in normal noisy environments.

#### 4.3.2. Camera-Based Results on SemanticOPV2V

We conduct experiments on SemanticOPV2V val set, as shown in Table 3. Following [37], we calculate IoU for each individual category and the mean IoU across all categories for occupancy and semantic occupancy prediction.

When focusing solely on binary occupancy prediction(as shown at Occ. Pred. in Table 3), we compare the method with Raw LiDAR baseline and CoHFF [37]. It is observed that TSMV has achieved the highest mean IoU metric and has led in many categories. In the final evaluation of semantic occupancy prediction(as shown at Sem. Occ. Pred. in Table 3), we further demonstrate the benefits brought by two-stream architecture. Our method achieves state-of-the-art results on the camera-based SSC task with an overall mean IoU increase of approximately 2.32%. As a result, the TSMV method can strengthen collaborative semantic scene completion tasks.

### 4.4. Run Time Efficiency

The real-time performance is displayed in Table 4. Notably, the time we calculate here only includes inference time on the ego vehicle. We tested the real-time performance on NVIDIA RTX 3060Ti. The results demonstrate that the proposed approach achieves competitive running efficiency and state-of-the-art model performance.

### 4.5. Detection Visualization

**Figure 4:**
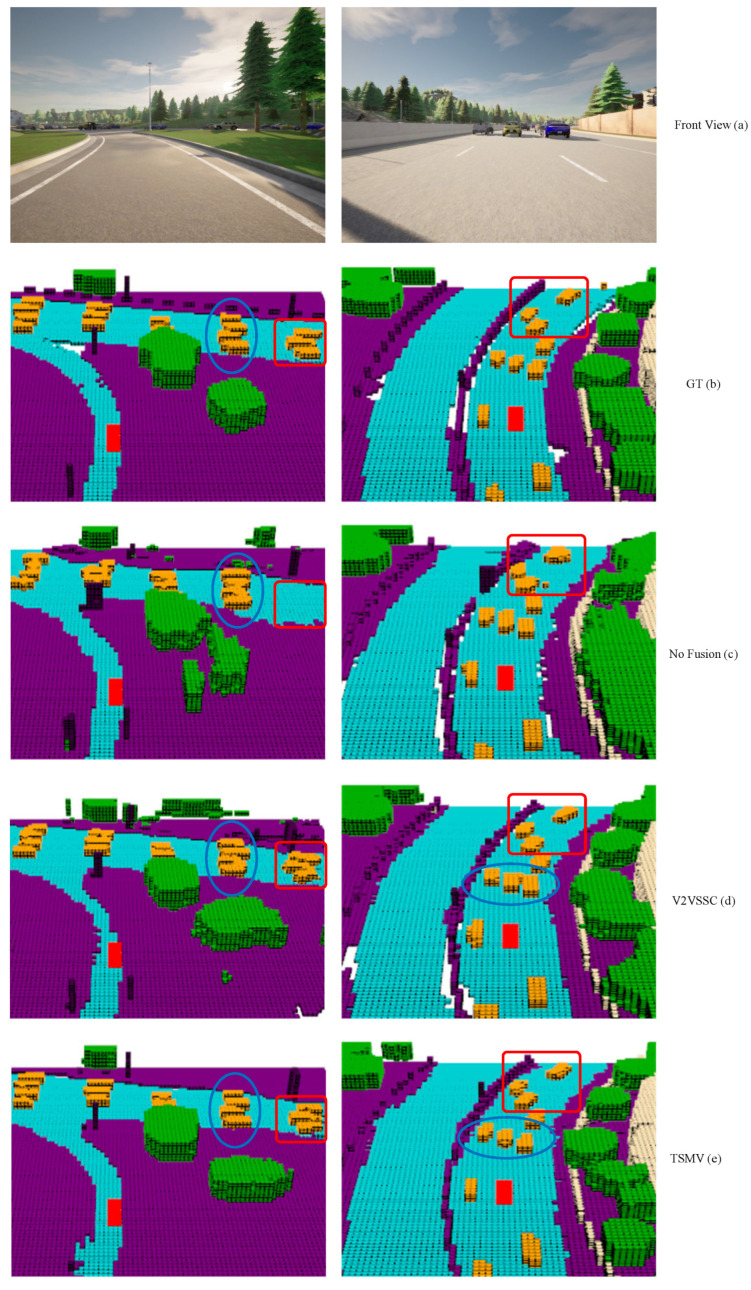
Visualization result. The red area in the figure represents the location of the ego vehicle.

Figure 4 shows the ego vehicle’s front view (a), semantic occupancy ground truth (b), and the detection visualization of No Fusion (c), V2VSSC (d), TSMV (e) method. Our model can predict the occupancy of 3D semantic scenes more accurately and align with the ground truth better. Compared with the method of no-fusion, the multi-vehicle cooperative method can better deal with some occluded areas. For example, where the red box is marked in the picture, from the front view, we can not observe these AVs, but the different perspectives brought by other vehicles can solve the problem of single-view occlusion. TSMV can more accurately describe the boundaries of each category, such as the blue box marked out, no-fusion, and V2VSSC method will show adhesion to adjacent vehicles.

**Figure 5:**
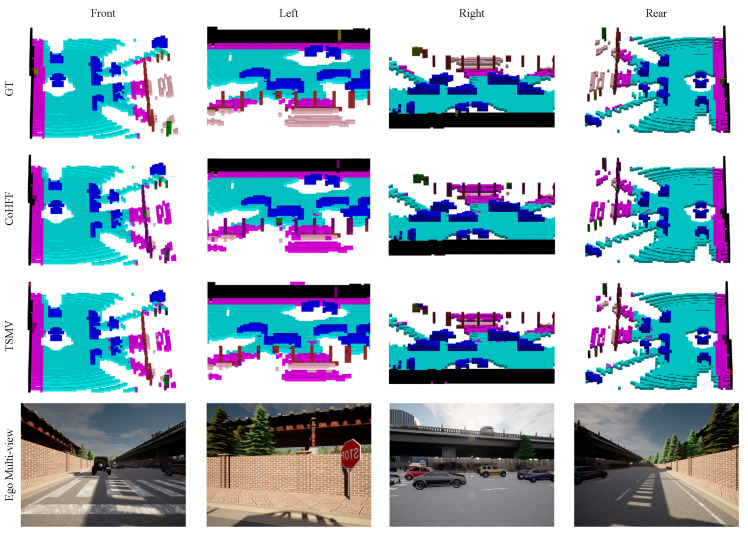
Illustration of collaborative semantic occupancy prediction from multiple perspectives, compared to the ground truth.

Figure 5 presents visual results of the CoHFF and TSMV model in SemanticOPV2V, which are compared with the ground truth data from multiple perspectives. All models can accurately predict various categories of voxels, such as roads, sidewalks, and vehicles. Our TSMV is better than the CoHFF model in terms of overall building prediction(red voxel in the picture) and is closer to the ground truth.

### 4.6. Ablation Studies

#### 4.6.1. Contribution of Major Components

We investigate the effectiveness of each component of TSMV on the V2VSSC dataset. Ablation experiments are performed on the two-stream architecture and NSCAT module proposed in this study. Our base model is a single vehicle baseline that only uses the ego stream and we compare the method that only uses the fuse stream. Then, we assess the influence of each component by incrementally incorporating a two-stream structure and an NSCAT module. As shown in Table 5, the proposed two-stream architecture and NSCAT module are conducive to performance improvement which proves the effectiveness of the two components. The two-stream architecture and NSCAT module increase mIoU by 2.1% and 2.7% respectively.

#### 4.6.2. Impact of the Kernel Size in the Neighborhood

Here we examine the impact of the kernel size of the neighborhood on TSMV in the V2VSSC dataset. As outlined in Table 6, enlarging the kernel size can effectively expand the global receptive field, particularly benefiting larger object categories (e.g., Road, Terrian, and Vegetable). Conversely, reducing the kernel size enhances the precision in localizing smaller object categories (e.g., Car and Pole), leading to improved performance. Thus, the choice of the appropriate kernel size of the neighborhood necessitates consideration of both global and local receptive fields. Finally, we choose nine as the kernel size to better balance the accuracy of each category.

#### 4.6.3. Performance of Positional Error

**Figure 6:**
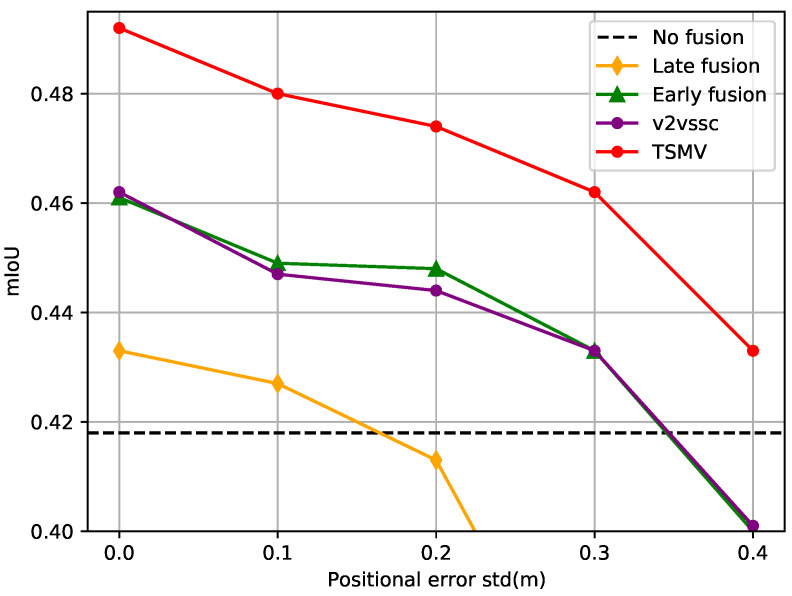
Positional error performance. The abscissa represents the standard deviation of the positioning sampling noise of the Gaussian distribution, and the ordinate represents the mIoU metric.

We quantify the level of noise via positional errors separately. The Gaussian distribution is utilized for sampling noise with the standard deviation $\sigma_{xyz}\in \left[0,0.4\right]$ m. As illustrated in Figure 6, TSMV achieves a significant advantage over other models when the positional discrepancy remains within the normal range ($\sigma_{xyz}\leq 0.2$ m [63,64]). Even with substantial noise ($\sigma_{xyz}=0.4$ m, twice higher than the maximum normal noise value [63,64]), TSMV outperforms the no-fusion baseline model, while the remaining models fall below the performance of the no-fusion baseline model, indicating our model possesses certain anti-interference capabilities.

## 5. Discussion

In the previous section, this study analyzed five experiments conducted to evaluate the performance of our proposed collaboration approach, TSMV. The following section is delved into these experimental results’ theoretical and practical significance. It will also discuss this study’s limitations and future research directions.

The results of Experiment One demonstrates that TSMV performs well across all metrics in both perfect and normal noisy environments, highlighting the potential of the two-stream architecture in feature fusion and the reliability of TSMV in normal noisy settings. Experiment Two demonstrates that TSMV can enhance collaborative semantic scene completion tasks in camera-based approaches. Experiment Three demonstrates that our proposed method balances performance and efficiency. Experiment Four, through visualization, illustrates the superiority of the multi-vehicle collaborative method in semantic scene completion, with TSMV providing more precise object localization and semantic segmentation within the collaborative approaches. Experiment Five demonstrates the effectiveness of components in TSMV. The two-stream architecture improves performance, with further enhancements from the NSCAT module. Experiment Six demonstrates the neighborhood size also impacts the performance. Larger neighborhoods improve performance for larger object categories, while smaller neighborhoods benefit smaller object categories by concentrating receptive fields within a smaller spatial range. Thus, selecting an appropriate neighborhood size can better balance performance across different categories. Experiment seven, through the quantification of noise, illustrates our proposed method presents a certain anti-interference capacity within a certain range of noise. These results not only validate the feasibility of our proposed method but also strongly support the applicability of collaborative perception in semantic scene completion in autonomous driving scenarios. The experimental results of TSMV not only enhance our understanding of the performance of collaborative perception in semantic scene completion but also guide the collaborative perception feature fusion method.

Despite significant advancements, TSMV has some limitations. The framework relies heavily on robust V2V communication, which may face reliability challenges with poor connectivity or network failures. To validate the influence of poor connectivity, we randomly delay signals obtained from other vehicles by different time ranges. The results are shown in Table 7. Network failure dramatically degrades the model performance for all V2V methods, but is still better than the no-fusion baseline. In addition, the extreme noise results in positioning errors, which leads to feature misalignment and restricts its application in highly adverse circumstances. Despite a considerable performance decline in extreme noise environments, the performance of TSMV can still yield the best result (and above the no-fusion setting) within a certain noise range. We will carry out further optimization in the future.

## 6. Conclusions

In this paper, we propose a novel two-stream multi-vehicle collaboration approach (TSMV) to address the ambiguity issue caused by feature misalignment between cooperative vehicles. The key component is a Neighborhood Self-Cross Attention Transformer module (NSCAT), which enables interactive learning and fusion of two-stream features at a reduced time cost. Features learn global environmental information from the fuse stream while retaining the accurate environmental boundary information from the ego stream which alleviates the boundary ambiguity issue and achieves collaborative perception. Comprehensive experiments in collaborative V2VSSC and SemanticOPV2V datasets showed that TSMV improves the performance of collaborative SSC tasks and achieves state-of-the-art results. In the future, we plan to test and validate our method in real-world scenarios to further evaluate its stability and robustness.

## Figures and Tables

**Table 1:** Semantic scene completion performance comparison on V2VSSC test set in perfect settings in detail. Bold font indicates the best on the column.

Method	IoU	mIoU	cIoU	Road	Car	Terrian	Building	Veg. ^1^	Pole
No Fusion	0.512	0.418	0.604	0.643	0.566	0.464	0.324	0.383	0.126
Late Fusion	0.54	0.433	0.608	0.67	0.546	0.478	**0.368**	0.419	0.115
Early Fusion	0.57	0.461	0.649	0.682	0.616	0.508	0.34	0.444	0.174
Fcooper [40]	0.556	0.455	0.64	0.676	0.604	0.513	0.323	0.44	0.172
V2XViT [26]	0.58	0.44	0.58	0.658	0.501	0.558	0.359	0.433	0.131
V2VSSC [25]	0.566	0.462	0.658	0.672	0.644	0.499	0.336	0.446	0.177
**TSMV(Ours)**	**0.586**	**0.492**	**0.687**	**0.708**	**0.666**	**0.577**	0.338	**0.481**	**0.18**

^1^ Veg. is the abbreviation of Vegetation.

**Table 2:** Semantic scene completion performance comparison on V2VSSC test set in noisy settings. Bold font indicates the best on the column in noisy settings.

Method	Perfect			Noisy		
	IoU	mIoU	cIoU	IoU	mIoU	cIoU
No Fusion	0.512	0.418	0.604	0.512	0.418	0.604
Late Fusion	0.54	0.433	0.608	0.526	0.403	0.544
Early Fusion	0.57	0.461	0.649	0.559	0.441	0.613
FCooper [40]	0.556	0.455	0.64	0.552	0.447	0.626
V2XViT [26]	0.58	0.44	0.58	0.57	0.431	0.565
V2VSSC [25]	0.566	0.462	0.658	0.559	0.443	0.618
**TSMV(Ours)**	**0.586**	**0.492**	**0.687**	**0.572**	**0.468**	**0.648**

**Table 3:** Semantic occupancy prediction on SemanticOPV2V val set. Bold font indicates the best on the line of different prediction tasks.

Task Type	Occ Pred.			Sem. Occ. Pred.	
**Method**	**RL ^1^**	**CoHFF [37]**	**TSMV**	**CoHFF [37]**	**TSMV**
mean IoU	57.12	65.24	**65.76**	31.72	**34.04**
Building (5.40%)	**67.5**	44.08	43.63	24.00	**31.49**
Fence (0.85%)	59.40	63.63	**64.15**	22.86	**27.71**
Terrain (4.80%)	43.60	76.99	**77.37**	10.21	**12.89**
Pole (0.39%)	**66.3**	61.57	61.29	46.33	**49.93**
Road (40.53%)	51.47	87.9	**88.68**	64.80	**65.80**
Side walk (35.64%)	45.46	86.27	**86.49**	57.44	**63.11**
Vegetation (1.11%)	**43.61**	33.26	33.94	10.47	**10.86**
Vehicles (9.14%)	41.4	70.15	**72.62**	82.21	**84.11**
Wall (2.01%)	71.51	80.48	**81.13**	**38.23**	37.74
Guard rail (0.04%)	**49.67**	39.91	39.76	**23.81**	17.97
Traffic signs (0.05%)	**68.98**	57.06	58.1	0.24	**6.93**
Bridge (0.04%)	76.53	81.59	**81.97**	0.00	0.00

^1^ RL(Raw LiDAR) is used as a baseline for the evaluation on the task of occupancy prediction.

**Table 4:** Inference time measured on GPU NVIDIA RTX 3060Ti.

Method	Parameter (M)	Time (ms)	mIoU (prf)
No Fusion	10.03	50	0.418
Late Fusion	10.03	50	0.433
Early Fusion	10.03	54	0.461
V2XViT [26]	15.07	88	0.44
V2VSSC [25]	10.03	31	0.462
TSMV(Ours)	11.23	48	0.492

**Table 5:** Component ablation study. Two-stream represents two-stream architecture, and NSCAT represents the Neighborhood Self-Cross Attention Transformer module.

Two-Stream		NSCAT	mIoU (prf)
Ego Stream	Fuse Stream		
✓	×	×	0.418
×	✓	×	0.444
✓	✓	×	0.465
✓	✓	✓	0.492

**Table 6:** Kernel size of neighborhood ablation study. Bold font indicates the best on the column.

Size	mIoU	Road	Car	Terrian	Buiding	Vegetable	Pole
3	0.475	0.7	**0.673**	0.528	0.303	0.462	**0.183**
5	0.477	0.709	0.662	0.532	0.334	0.461	0.17
7	0.48	0.711	0.658	0.563	**0.347**	0.466	0.132
9	**0.492**	0.708	0.666	0.577	0.338	0.481	0.18
11	0.486	**0.715**	0.653	**0.586**	0.333	**0.487**	0.143

**Table 7:** Performance of different transmission delays.

Method	0	0–500	0–1000	0–2000	0–3000
No Fusion	0.418	0.418	0.418	0.418	0.418
Late Fusion	0.433	0.414	0.403	0.398	0.397
Early Fusion	0.461	0.416	0.404	0.397	0.395
V2VSSC [25]	0.462	0.427	0.415	0.409	0.406
TSMV	0.492	0.452	0.44	0.435	0.433

## Data Availability

Data available on request due to restrictions.
